# Longitudinal Stroke Recovery Associated With Dysregulation of Complement System—A Proteomics Pathway Analysis

**DOI:** 10.3389/fneur.2020.00692

**Published:** 2020-07-28

**Authors:** Vinh A. Nguyen, Nina Riddell, Sheila G. Crewther, Pierre Faou, Harinda Rajapaksha, David W. Howells, Graeme J. Hankey, Tissa Wijeratne, Henry Ma, Stephen Davis, Geoffrey A. Donnan, Leeanne M. Carey

**Affiliations:** ^1^Department of Occupational Therapy, La Trobe University, Bundoora, VIC, Australia; ^2^Department of Psychology and Counselling, La Trobe University, Bundoora, VIC, Australia; ^3^Neurorehabilitation and Recovery, Stroke, The Florey Institute of Neuroscience and Mental Health, Heidelberg, VIC, Australia; ^4^Western Health, Department of Neurology, Sunshine, VIC, Australia; ^5^Department of Biochemistry and Genetics, La Trobe University, Bundoora, VIC, Australia; ^6^Medical Sciences Precinct, University of Tasmania, Hobart, TAS, Australia; ^7^Faculty of Health and Medical Sciences, Internal Medicine, University of Western Australia, Perth, WA, Australia; ^8^Clinical Research, Harry Perkins Institute of Medical Research, Perth, WA, Australia; ^9^Department of Medicine, The University of Melbourne, Sunshine, VIC, Australia; ^10^Monash Health, Neurology and Stroke, Clayton, VIC, Australia; ^11^Department of Neurology, Royal Melbourne Hospital, Parkville, VIC, Australia

**Keywords:** longitudinal, stroke, proteomics, immune system, complement system, bioinformatics, systems biology, pathway analysis

## Abstract

Currently the longitudinal proteomic profile of post-ischemic stroke recovery is relatively unknown with few well-accepted biomarkers or understanding of the biological systems that underpin recovery. We aimed to characterize plasma derived biological pathways associated with recovery during the first year post event using a discovery proteomics workflow coupled with a topological pathway systems biology approach. Blood samples (*n* = 180, ethylenediaminetetraacetic acid plasma) were collected from a subgroup of 60 first episode stroke survivors from the Australian START study at 3 timepoints: 3–7 days (T1), 3-months (T2) and 12-months (T3) post-stroke. Samples were analyzed by liquid chromatography mass spectrometry using label-free quantification (data available at ProteomeXchange with identifier PXD015006). Differential expression analysis revealed that 29 proteins between T1 and T2, and 33 proteins between T1 and T3 were significantly different, with 18 proteins commonly differentially expressed across the two time periods. Pathway analysis was conducted using Gene Graph Enrichment Analysis on both the Kyoto Encyclopedia of Genes and Genomes and Reactome databases. Pathway analysis revealed that the significantly differentiated proteins between T1 and T2 were consistently found to belong to the complement pathway. Further correlational analyses utilized to examine the changes in regulatory effects of proteins over time identified significant inhibitory regulation of clusterin on complement component 9. Longitudinal post-stroke blood proteomics profiles suggest that the alternative pathway of complement activation remains in a state of higher activation from 3-7 days to 3 months post-stroke, while simultaneously being regulated by clusterin and vitronectin. These findings also suggest that post-stroke induced sterile inflammation and immunosuppression could inhibit recovery within the 3-month window post-stroke.

## Introduction

Ischemic stroke covers a variety of cerebrovascular events that affect up to 800,000 people in the United States every year, with 133,000 deaths reported in 2017 (16.74%) (1). Of the survivors, 30% are reported to experience prolonged cognitive impairment (2) and depressive symptoms at any point 5 years post-stroke (3). Currently there are few well-accepted biomarkers for recovery and comparatively little literature exploring the biological systems that drive recovery or even the most optimal times for monitoring biological and behavioral recovery. Evidence from stroke rehabilitation studies suggest the greatest efficacy for motor-based rehabilitation is within this 3 month time window (4), though recovery may continue at a slower rate over subsequent months and years. Although there has been increasing research examining the blood biomarkers of stroke recovery (5), the additional linking of biomarkers to biological systems remains speculative. Hence, we aimed to investigate the changes in the molecular profile of proteins in plasma samples via a mass spectrometry (MS) based discovery proteomics approach (6). Mass spectrometry and nuclear magnetic resonance (NMR) based techniques examining protein expression are among the most versatile techniques for protein identification and quantification, with the ability to address a wide range of biological samples, especially plasma, and serum (7, 8). Proteomics utilizes the advantage of systems biology techniques to quantify a large number of analytes in an exploratory fashion, with a computational bioinformatics approach to further categorize biomarkers into biosystems (9).

Proteomics have recently been used to pursue multiple clinical questions within stroke research, relating to differentiation of ischemic from hemorrhagic stroke (10–12) and investigations of potential biomarkers involved in post-stroke recovery (13, 14). Although traditional bioinformatics methods were originally developed to accommodate gene expression data, proteomics studies can utilize these methods to organize and visualize findings by adopting standardized change scores and using annotations that are common across proteomics and genomics (15). Indeed, our laboratory has previously used proteomic methods and Gene Set Enrichment Analysis (GSEA) to investigate the relationship between protein changes in plasma at 3-months post-stroke and affective (depression) outcomes (16). The results indicated that proteins involved in the complement but not the coagulation pathway of the immune system are likely to be associated with post-stroke depressive symptoms (14). The complement system is recognized as an innate immune pathway that contributes to primary host defence by encouraging phagocytosis of unwanted cells. This new study aims to expand upon our earlier single time point study by using discovery proteomics to identify longitudinal changes in blood plasma protein expression over the post-stroke timeline of recovery; specifically 3 timepoints post ischemic stroke: 3–7 days (T1), 3-months (T2) and 12-months (T3). We also aimed to improve upon our previous set-based functional annotation methods by utilizing Gene Graph Enrichment Analysis (GGEA). The GGEA approach differs from the GSEA approach by further addition to traditional set-based functional annotations by incorporation of *networks* of established biochemical relationships (gene to gene) using topological omics databases such as Reactome (17) and KEGG (18) for the analysis of structural representation of biological pathways in the analytical workflow. This more novel approach further addresses the regulatory mechanisms in gene and protein pathways by examining co-expression and co-regulation networks using correlation analyses (19). Examining the changes in correlational strength also allows for quantification of the changes in the regulatory effect of proteins between timepoints. Although discovery approaches are hypothesis-free by nature, based on our previous study (14) and others (20) that suggest inflammatory and immune homeostasis will be disrupted in the post-stroke recovery timeline, we hypothesize that the complement system will be dysregulated when comparing early 3–7 days post-stroke to later 3-month and 12–month post-stroke timepoints.

## Materials and Methods

### Subjects

Data from a subset of ischemic stroke patients was obtained from the longitudinal stroke cohort known as START, which comprised participants from START_PrePARE (STroke imAging pRevention and Treatment: Prediction and Prevention to Achieve optimal Recovery Endpoints; (21) (Neuroscience Trials Australia: NTA 0902) and START_EXTEND [STroke imAging pRevention and Treatment: EXtending the time for Thrombolysis in Emergency Neurological Deficits (22) (Neuroscience Trials Australia: NTA 0901, Clinicaltrials.gov number: NCT01580839)] studies. These prospective, integrated studies were longitudinal and provided long term plasma and serum samples at 3 time-points post-stroke. Selection of the subset ischemic stroke patients for the current study (*n* = 106) was initially based on the availability of ethylenediaminetetraacetic acid (EDTA) treated plasma samples at each of the 3 timepoints: 3–7 days (T1), 3-months (T2) and 12 months (T3). Laboratory batch processing limitations and time/cost considerations allowed for 180 EDTA plasma samples to be processed, i.e., samples from 60 stroke patients across 3 timepoints. We therefore conducted a stratified selection process involving patients with complete blood samples at the 3 repeated times and full clinical scores, to ensure a spread of scores across clinical outcomes of stroke severity, mood, and cognition. As expected, the current subset contained only participants who completed the 3 sessions in the 12 months post first acute stroke event. See Table 1 below for baseline characteristics for patients (43 male) and Table 2 for clinical characteristics at T1, T2, and T3. The total START_PrePARE and START_EXTEND cohort comprised 219 patients, with 21 deaths, 8 lost to follow-up and 2 withdrawals. See Supplementary Table 1 for a comparison between the selected subset and the total cohort. Healthy control data was not available as part of the original protocols for the START_PrePARE and START EXTEND studies. Ethics was approved by the Human Research Ethics Committee of Austin Hospital, Heidelberg (HREC code: H2010/03588), and relevant university and hospital sites.

**Table 1:** Baseline sample characteristics (*n* = 60).

**Baseline**	**Mean**	**SD**	**Median**	**IQR**
Age (years)	68.00	14.60	69.00	18.00
NIHSS	4.70	4.76	2.50	3.00
Height (cm)	160.41	26.80	170.00	20.00
Weight (kg)	74.66	19.80	80.00	26.75
Heart rate (per minute)	74.52	11.06	74.50	14.50
Systolic blood pressure (mm Hg)	141.47	23.27	134.50	29.75
Diastolic blood pressure (mm Hg)	78.50	11.77	78.00	14.00
**TOAST Criteria**	**Frequency**	**Percentage**		
Large artery atherosclerosis	14	24.14%		
Cardioembolism	6	10.34%		
Small vessel occlusion	15	25.86%		
Stroke of other determined etiology	3	5.17%		
Stroke of undetermined etiology, two or more causes identified	3	5.17%		
Stroke of undetermined etiology, negative evaluation	3	5.17%		
Stroke of undetermined etiology, incomplete evaluation	14	24.13%		
**Comorbidities**				
Past atrial fibrillation	4	6.7%		
Hypertension	26	43.3%		
Lipid disorder	24	40.0%		
Ischemic heart disease	11	18.3%		
Diabetes mellitus	9	15.0%		

*n = 60; NIHSS, National Institute of Health Stroke Scale; 0, No Stroke symptoms; 1–4, Minor Stroke; 5–15, Moderate Stroke; 16–20, Moderate to Severe Stroke; 21–42, Severe Stroke (73); TOAST, Trial of Org 10172 in Acute Stroke Treatment classification for ischemic stroke subtype*.

**Table 2:** Sample Clinical Characteristics at 3–7 days, 3 months and 12 months (*n* = 60).

	**3–7 Days (T1)**				**3 Months (T2)**				**12 Months (T3)**			
	**Mean**	**SD**	**Median**	**IQR**	**Mean**	**SD**	**Median**	**IQR**	**Mean**	**SD**	**Median**	**IQR**
Weight (kg)					76.30	15.30	78.00	19.00	78.75	13.57	79.50	20.00
Heart rate (per minute)					63.02	21.03	65.00	20.00	61.50	21.01	70.00	19.00
Systolic blood pressure (mm Hg)					125.76	16.92	125.50	21.00	124.28	15.34	124.00	17.25
Diastolic blood pressure (mm Hg)					74.78	10.17	76.00	11.75	72.47	9.78	70.00	15.00
NIHSS	2.35	3.05	1.00	3.00	1.18	2.31	0.00	1.00	2.45	12.87	0.00	1.00
mRS^*^					1.25	1.31	1.00	2.00	1.12	1.22	1.00	2.00
MoCA	24.21	5.00	26.00	5.00	25.93	4.57	28.00	5.00	25.07	5.02	26.00	4.00
MADRS	6.93	7.31	4.00	11.00	8.72	8.81	5.50	13.75	7.13	7.41	5.00	10.75

*NIHSS, National Institute of Health Stroke scale; mRS, modified Rankin Scale; MADRS, Montgomery-Åsberg Depression Rating Scale; MoCA, Montreal Cognitive Assessment*.

* *The mRS is not conducted at 3–7 days as it is a measure of post-stroke disability within previous 30 days*.

### Blood Collection and Storage

All samples were collected in Benton-Dickson (BD) EDTA-coated 4 ml vacutainers and were mixed and left to stand in ambient room temperature for 30 min. Average time to blood draw was 3.45 days (range = 2.03–7.58 days, SD = 1.174) after stroke onset for T1. The tubes were then centrifuged at 1100–1300 g at room temperature and the resulting plasma was aliquoted into cryotubes and immediately stored in a −80^o^C freezer. For transport from the central study freezer to the analysis site, temperature was kept at −70 to −80^o^C on dry ice before transfer into a −80^o^C freezer. This procedure was consistent for the 3-month and 12-month follow up periods.

### Mass Spectrometry

Label-free quantitation (LFQ) proteomics was conducted on the Q Exactive HF Orbitrap instrument (Thermo-Fisher Scientific). Details of the sample preparation, instrument parameters and protein identification are available in the Supplementary Methods.

### Bioinformatics and Statistical Analysis

The initial MaxQuant output consisted of 358 identified proteins across 180 samples. After removal of site-identified contaminants and filtering for at least 80% of data present across the protein, 163 proteins remained (Supplementary Data 1). This procedure regarding missing values is an expected step in addressing proteomics data as the protein identification search on MaxQuant (utilizing an FDR of 1%) will not output as an identified protein in the final data file unless there is a single unique peptide, regardless of peptide length. The Limma R package (version 3.34.9) with Benjamini–Hochberg (BH) adjustments for familywise error rate was used to compare which proteins were differentially expressed across T1, T2, and T3. To further understand the functional organization of significant protein sets, the EnrichmentBrowser R package (version 2.09.17) was employed with GGEA as the network-based enrichment method across the KEGG and Reactome databases using default settings (minimum set size = 3, maximum set size = 500, permutations = 1000). Correlations with BH corrections were used to further explore the changes in proteins that regulate identified pathways, with Fisher *r* to *z* transformations conducted to explore changes in regulatory effect across timepoints. Statistical significance levels were set to *a* =.05 after multiple comparisons adjustment.

### Data Availability

Due to the requirements of ethics and the nature of ongoing clinical trials with the START cohorts, unidentified patient clinical data may only be made available upon request. The mass spectrometry proteomics data have been deposited to the ProteomeXchange Consortium via the PRIDE (23) partner repository with the dataset identifier PXD015006.

## Results

The differential expression (DE) analysis revealed that 29 proteins significantly differed between T1 and T2, and that 33 proteins significantly differed between T1 and T3 (Table 3) with 8 proteins at FDR of 0.05 indicating that the effects are higher than would occur due to chance alone. The changes between T1 to T2 and T1 to T3 constitute 17.79 and 20.25%, respectively, in proportion to the total number of proteins identified. Eleven proteins were uniquely expressed between T1 and T2 and 15 proteins were uniquely expressed between T1 and T3, with 18 of the same proteins expressed both between T1 and T2 and T1 and T3. There were no significant differences in protein expression between T2 and T3, potentially suggesting that the currently identified proteome in post-stroke survivors does not change significantly between 3- and 12-month times. See Supplementary Data 1 for a full list of proteins and fold change values across all comparisons.

**Table 3:** Differentially expressed proteins (BH *p* < 0.05) detected between T1, T2 and T1, T3.

**3–7 Days (T1) to 3 Months (T2)**			**3–7 Days (T1) to 12 Months (T3)**		
**Gene symbol**	**log2 fold change**	**adj *p***	**Gene symbol**	**log2 fold change**	**adj *p***
C8A	0.6657	0.0000	C8A	0.7394	0.0000
ACTB	0.3766	0.0000	APOA4	0.3585	0.0000
APOA4	0.3169	0.0000	ACTB	0.3712	0.0000
A1BG	−0.3210	0.0004	CFI	0.3346	0.0000
C9	−0.2814	0.0027	APOA2	0.2349	0.0003
CLEC3B	0.1814	0.0027	C9	−0.3231	0.0003
FBLN1	0.2953	0.0044	PGLYRP2	0.1863	0.0006
CFI	0.2417	0.0086	CLEC3B	0.1992	0.0006
APOD	0.1584	0.0104	APOB	−0.1754	0.0049
PGLYRP2	0.1634	0.0104	CFD	0.2459	0.0065
APOF	−0.2793	0.0109	CPN1	−0.1435	0.0065
IGFALS	0.1975	0.0131	TF	0.2243	0.0065
F9	−0.2180	0.0133	LRG1	−0.2285	0.0066
SERPINA3	0.1332	0.0133	RBP4	0.2153	0.0067
SAA2-SAA4	−0.2462	0.0141	FGA	0.1504	0.0068
RBP4	0.1836	0.0146	APOD	0.1399	0.0068
TF	0.2026	0.0146	A1BG	−0.2353	0.0073
APOC1	−0.1699	0.0286	IGFALS	0.1906	0.0073
FETUB	0.1995	0.0286	SAA2-SAA4	−0.2728	0.0081
HPR	−0.1673	0.0295	A2M	−0.1860	0.0095
PLTP	0.3322	0.0295	AFM	0.1487	0.0160
SAA1	−0.9248	0.0295	C4BPB	−0.2011	0.0160
FGB	−0.2347	0.0308	CSPG4	0.2386	0.0160
APOB	−0.1347	0.0389	C4BPA	0.1206	0.0187
C8G	−0.1511	0.0389	APCS	−0.2772	0.0198
VTN	0.0723	0.0413	VTN	0.0737	0.0244
SERPINA10	−0.2143	0.0420	GPLD1	0.2335	0.0274
HPX	0.0918	0.0433	ORM1	−0.3497	0.0274
C6	0.0980	0.0493	LBP	−0.2497	0.0282
			FBLN1	0.2117	0.0296
			SAA1	−0.9380	0.0334
			SERPINA3	0.1000	0.0374
			HPX	0.0898	0.0440

*p < 0.05 adjusted by the Benjamini–Hochberg procedure was considered statistically significant. Lists are ordered by p-value*.

The list of DE proteins and the full expression matrix were submitted to EnrichmentBrowser using GGEA as the network enrichment method (1000 permutations and α = 0.05). This revealed significant functional annotation of our set of proteins in both KEGG and Reactome databases only between T1 and T2, after BH adjustment of *p*-values (Table 4) (see Supplementary Data 2 a full list of nominally significant sets from KEGG and Reactome). In the case of the Reactome database, the nested structures in the ontological organization of the pathways can lead to redundancy resulting in identification of multiple pathways, especially as the “Complement Cascade” is located as a subset of the “Innate Immune System” and the “Immune System.” Further interpretation is needed to account for the number and type of identified proteins between the reference pathways and experimental data and should occur at the level closest to the relevant biological processes. Both KEGG and Reactome databases concurred on the identification of the complement system as the significantly enriched network in these plasma samples.

**Table 4:** Significantly Enriched Gene Pathways between T1 (3–7 days) and T2 (3-month) timepoints in Stroke Survivors.

**Gene Set**	**Identifier**	**Normed Score**	**adj *p***
**KEGG**			
Complement and coagulation cascades	hsa04610	1.91	0.003
**Reactome**			
Immune system	R-HSA-168256	0.497	0.0009
Innate immune system	R-HSA-168249	0.497	0.0009
Complement cascade	R-HSA-166658	0.497	0.0009

*p < 0.05 adjusted by the Benjamini–Hochberg procedure was considered statistically significant*.

**Figure 1:**
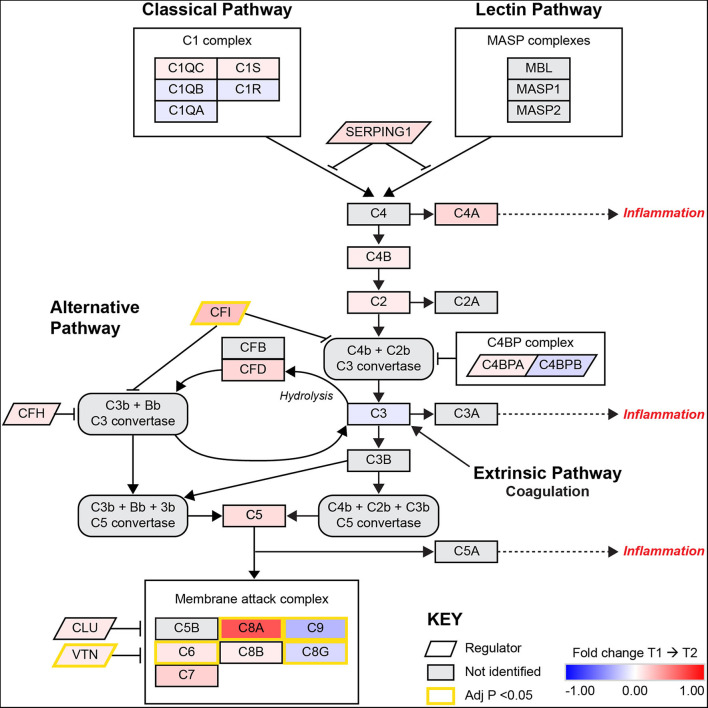
A pathway diagram contrasting information from differential proteomic analysis of KEGG and Reactome GGEA complement system pathways at 3–7 days (T1) and 3-months (T2) post-stroke. This pathway is activated by the C1q complex antigen linked immunoglobulin through the classical pathway and pathogen surface linked mannose binding linked (MBL) proteins through the MASP complex. The alternative pathway of complement activation follows a constant and low state of activation by a feedback loop that is heavily regulated by complement factor I (CFI) and complement factor H (CFH). Simultaneous upregulation of complement factor D (CFD) and CFI suggest that the alternative pathway is undergoing activation but also being regulated to prevent autoimmune insult. Recently, the link between coagulation and complement has been established through thrombin, plasmin and factor XIIa, these factors were not identified or significantly related to complement in this model. All activation pathways lead to the promotion of cleavage of fluid phase complement component 3 (C3) in the bloodstream, with heavier β chains forming convertases downstream and lighter α chains such as C3a and complement component 5α (C5a) able to signal for potent local and systemic inflammation responses, with the most extreme being anaphylactic shock. The final stage of complement activation is the membrane attack complex (MAC), a large protein complex that is constructed on cells to disrupt the outer membrane and promotes active cytolysis. The profile of significant differences in MAC proteins such as C8G and C9 suggest plasma complement regulation by vitronectin (VTN) or clusterin (CLU). Image adapted from KEGG [map04610: Complement and coagulation cascades (homo sapiens)] with permission.

A visual illustration of this enriched pathway on the Reactome database reveals the changes and interactions of the detected molecules on the complement pathway (Supplementary Figure 1), displaying information on nodes (proteins) and edges (the correlation between nodes). Similarly, the results from the KEGG database includes elements of the complement pathway but also the coagulation cascades (Supplementary Figure 2). As the complexity of information is difficult to interpret without a significant degree of system specific understanding in these pathway diagrams, a composite network pathway was created to amalgamate the statistical relationships found in both diagrams pertaining to the complement system as shown in Figure 1.

Some of the regulatory proteins in the complement pathway (CLU & VTN) were not included in the pathway diagrams for KEGG and Reactome databases used in this analysis. Therefore, a correlation matrix with BH corrected *p-*values was created to explore the direction and strength of the regulatory effects between regulator and related proteins (Table 5). Fisher's transformation of Pearson's *r* to normally distributed *z* scores was computed to compare changes in regulatory effects between T2 and T1. While not overall presenting significant results, examination of the *r*_diff_(T2-T1) complement regulators on their target proteins show that the middle stage complement regulators shifted their effects forward, resulting in a reduced inhibitory effect. Only the inhibitory effect of CLU on C9 was significantly increased from T1 to T2, with VTN also showing a large effect size for changes in C9 regulation.

**Table 5:** Pearson's Correlation matrix with Fisher *r* to *z* transformations for complement proteins and their regulators between T1 and T2.

**Stage**	**Regulator**	**Complement protein**	***r* (T1)**	***r* (T2)**	***r*_**diff**_ (T2-T1)**	***z*_**diff**_ (T2-T1)**	**adj *p***
Early	SERPING1	C1QA	−0.01	0.06	0.07	0.37	0.71
	SERPING1	C1QB	0.13	0.16	0.03	0.15	0.88
	SERPING1	C1QC	0.25	−0.01	−0.26	−1.44	0.15
	SERPING1	C1R	−0.11	0.00	0.12	0.62	0.53
	SERPING1	C1S	−0.18	−0.15	0.02	0.12	0.90
Mid	C4BPA	C4A	−0.03	0.17	0.20	1.08	0.28
	C4BPA	C4B	−0.08	0.08	0.16	0.86	0.39
	C4BPA	C2	0.04	0.06	0.01	0.08	0.94
	C4BPB	C4A	−0.13	0.17	0.31	1.64	0.10
	C4BPB	C4B	−0.15	−0.08	0.07	0.40	0.69
	C4BPB	C2	−0.26^*^	−0.05	0.21	1.13	0.26
	CFH	C3	0.37^*^	0.54^*^	0.16	1.10	0.27
	CFI	C3	0.15	0.27^*^	0.12	0.67	0.50
Late	CLU	C6	0.13	0.05	−0.07	−0.40	0.69
	CLU	C7	0.01	0.04	0.03	0.14	0.89
	CLU	C8A	0.10	−0.03	−0.13	−0.68	0.50
	CLU	C8B	0.35^*^	0.14	−0.20	−1.15	0.25
	CLU	C8G	−0.24	−0.18	0.06	0.31	0.76
	CLU	C9	−0.02	−0.49^*^	−0.47^**^	−2.74	0.01
	VTN	C6	0.25	0.19	−0.06	−0.32	0.75
	VTN	C7	0.23	0.10	−0.12	−0.68	0.49
	VTN	C8A	0.42^*^	0.28^*^	−0.14	−0.85	0.40
	VTN	C8B	0.24	0.29^*^	0.05	0.28	0.78
	VTN	C8G	0.10	0.15	0.04	0.24	0.81
	VTN	C9	0.04	−0.30^*^	−0.34	−1.87	0.06

The early stage of the complement pathway consists of activation units, the middle stage consists of protein activity related to cleaving C3 and C5, with the late stage consisting of proteins involved in membrane attack complex (MAC) formation. These correlations only show the effect of a single regulator on the related protein and does not take into consideration the downstream pathways. p was BH adjusted

* *p < 0.05*,

** *p < 0.01*.

## Discussion

Currently the interaction of the many biological mechanisms limiting post-stroke recovery remain poorly understood. To better understand the biochemical pathways impacting/affecting the post-stroke timeline of recovery, this study employed a discovery approach utilizing mass spectrometry to examine protein expression in patients at 3–7 days (T1), 3-months (T2), and 12-months (T3) post-stroke. Two sets of proteins were identified to be significantly different based on differential expression analysis from T1 to T2 and T1 to T3, but not between T2 and T3. Of these proteins, complement (C8A, C9, CFI), apolipoproteins (APOA4, APOA2, APOD) and membrane bound proteins (TF, ACTB) were highly overexpressed; consistent with high abundance typically found in human blood samples (24). The lists of significantly differently expressed proteins were analyzed using the GGEA bioinformatics algorithm based on the topological consistency of the observed data sets compared to database defined reference pathways. This revealed that the proteins identified in this longitudinal experiment significantly conformed to pathways central to the complement system in both KEGG and Reactome databases. To our knowledge, the current study is first published use of the GGEA approach in clinical proteomics of human blood.

There is currently limited knowledge of the changes in complement system in post-stroke recovery, especially relative to blood plasma concentrations in similar aged individuals (mean age 68 ± 14 years). Furthermore, most studies examining the complement system post-stroke have described its damage-exacerbating role in acute stroke human and animal models but have not examined changes in levels longitudinally over time post-stroke (25–29). Many of the studies exploring complement in stroke have examined the system in the context of danger associated molecular pattern (DAMP) signaling and subsequent neuronal repair in central nervous system (CNS) specific injury (30), with plasma and serum based studies examining levels in relation to clinical outcomes (31, 32). The endothelial junctions forming the blood brain barrier (BBB) have been traditionally viewed as preventative to the entry of large peripherally derived molecules such as complement into the cerebral parenchyma (33, 34). The CNS has also been recognized as able to endogenously biosynthesize complement proteins in glial cells (35). Although disruption to BBB permeability allows for the passage of complement proteins from periphery to CNS (34), to our knowledge, no study has simultaneously examined the levels and functional activity of the complement system in both domains. Therefore, the results of this study have been interpreted as referring to the circulating fluid phase proteins that enhance the decay of complement proteins by cleaving active proteins through allosteric binding (25).

The complement pathway has three traditional modes of activation, the classical, lectin and alternative pathways. The classical and lectin pathways are activated by antigen and pathogen binding complexes, respectively, whereas the alternative pathway is an internal biomechanical activation loop that regulates downstream pathway activity (36). In the classical pathway of complement activation, upregulation of SERPING1 or the C1 inhibitor protein is responsible for regulation of the C1 complex. Specifically, SERPING1 strongly binds to the C1r and C1s proteases and also to the activation units of the lectin pathway [mannose binding lectin, MBL, and associated proteases MASP1 and MASP2 (37)], effectively inhibiting the initial activation effects of the complement system in both antigen and pathogen related pathways. In addition to the effects on the complement system, high concentrations of this protease can further inhibit leukocyte-endothelial adhesion and vascular rolling via interference of cell adhesion molecules (CAMs) or selectins, the first physiological requirements for trans-endothelial leukocyte infiltration (38). Although a fold change of 0.008 or 0.8% for SERPING1 was detected between T1 and T2 in this study, previous reports have indicated that a fold change can be observed during acute inflammation and that higher levels may be therapeutic in preventing autoimmune injury by providing a stop mechanism to complement system activation (39, 40). These acute properties have been demonstrated in animal models of post-stroke middle cerebral artery occlusion (MCAO) and shown to attenuate ischemic reperfusion injury (41, 42). Limited evidence for longitudinal changes in levels of SERPING1 in human cardiovascular disease have previously been suggested as a link to low-grade levels of chronic inflammation (43). Furthermore, the incidence rates of post-stroke immunological trajectory of post-stroke recovery may also be explained by the immune inhibitory effects of SERPING1, following the initial phase of post-stroke immune flux and subsequent immunosuppression (44).

The cleavage of C3 is the central amplification step of the whole complement pathway, with active C3b forming the C5 convertase and C3a inducing further phagocytotic chemotaxis (29). Given that C3 is central in all complement activation pathways, especially with the alternative pathway requiring C3 hydrolysis, much of the theorized regulatory activity in this pathway is focused on inhibition of C3 convertase formation and decay rate (36). Of the complement regulatory proteins, CFH and CFI were overexpressed, with only plasma CFI levels shown to be statistically different. Additionally, the changes in regulatory effect for middle stage complement proteins show that even though CFH and CFI are upregulated between T1 and T2, their inhibitory effects were reduced. Although CFI and CFH are cofactors of C3 convertase (not identified in this study) inhibition and not C3 itself, the overall profile suggests a relative lack of effectiveness in the functional ability of these proteins to regulate alternative complement pathway activity in this older group. Furthermore, this profile may also be indicative of overexpression in levels of CD55 or decay accelerating factor (DAF), a protein that inhibits C3 and C5 convertase formation, albeit on cell surfaces (45). Complement C3a has previously been shown to be acutely elevated from 1 to 28 days post-stroke (46, 47). Indeed, ongoing anaphylatoxin signaling from components such as C3a and C5a are damaging to host systems, especially in cerebral ischemia (25). The quantification of properdin, a protein that acts as a positive regulator of C3 and C5 convertases (48), would further ameliorate understanding of alternative pathway activation in this sample.

Complement component C5 is the important functional unit of the pathway. Cleavage of soluble C5 protein into C5b is able to create the porous membrane attack complex (MAC) on targeted cells to induce cell death by cytolysis while C5a is able to produce local and system wide inflammatory cascades that are 20 times more potent than C3a (49). The profile here shows that C5 is upregulated at 3-months, despite reduced C3 levels. This suggests that the reduction in C3 levels may be indicative of active cleaving to produce downstream increased C3b and C3a levels to produce or maintain constant immune system homeostasis. The detection of increased C5 may also imply involvement of the novel complement pathway or the extrinsic complement pathway, linking coagulation, and complement systems. This pathway was originally thought to be activated based on thrombin functionally substituting for the C3 dependent C5 convertase (50). Recently, a study has also demonstrated that in thrombosis, interactions with plasmin in both surface bound and fluid phase complement is capable of upregulating C5a and C5b whether in venous or arterial thrombi (51). In our analysis however, plasminogen was not found to be significantly differentially expressed or related to the complement pathway (Supplementary Figure 2).

Our methodology identified and quantified all the molecular components in MAC formation pathway. Our findings demonstrate that C6 and C8A were significantly upregulated and C8G and C9 were significantly downregulated between T1 and T2. Regulators of MAC formation exhibit function by preventing polymerisation of the C8 or C9 complexes, thereby inhibiting cytolytic function on cell surfaces (52). Pore formation is heavily regulated by CD59 or MAC-inhibitory protein on host cell surfaces to prevent further immune insult resulting from early tissue induced hyperinflammation and gradual immune dysregulation (53). As this expression profile was obtained in plasma, the expression profile here is suggestive of fluid phase regulation of MAC formation, dependent on vitronectin (VTN or S-protein) and clusterin (CLU or apolipoprotein J). To create a successful MAC, from 12 to 16 molecules of C9 are needed as the final step to form a cyclical pore in addition to 1 of each previous MAC protein in the chain (54). Both VTN and CLU inhibit the formation of the MAC insertion C5b-7 complex and also inhibit the ability of C9 to bind to inserted MAC complexes to create functioning lysis pores (54). VTN and CLU were both found to be overexpressed, with the increases in VTN shown to be statistically significant (36). Overall, the profile of the complement system here appears to be dysregulated, with an increase in alternative pathway activation by CFB, and reduced inhibitory effectiveness of all middle stage complement proteins but especially CFI and CFH. The apparent contradiction in expected abundances of MAC proteins suggests that the pathway is also being inhibited by CLU and VTN.

Although few studies have presented this specific profile of complement expression, and currently none in a complex proteomics pathway model, there are some interpretations relevant to stroke survival and recovery that can be made here. From the upregulation of C5 and initial MAC proteins, it is possible that C5 is being continually cleaved to produce the pro-inflammatory chemokine C5a. This can be understood in theory of post-stroke immune profiles where both central and peripheral systems never fully recover to pre-stroke levels and establish new albeit dysfunctionally higher inflammation profiles (44).

The profile of complement activation described in this study is likely to indicate that

1. the internal activation loop that readies the immune system for pathological threats and tissue damage (the alternative pathway) is activated,
2. middle stage complement regulators that are normally expected to be activated to prevent indiscriminatory autoimmune damage are overexpressed but their intended inhibitory effects (CFI –> C3) are reduced and
3. although early chain MAC proteins (C6, C7, C8A, C8B) trend toward overexpression by time T2 are likely due to alternative pathway activation, CLU and VTN are also overexpressed, possibly as a compensatory mechanism for alternative pathway activation and thereby preventing autoimmune MAC formation by inhibiting C9 expression on host/friendly cells.

Our results did not find/demonstrate an association between C8G expression and CLU or VTN, although downregulation of C8G is theorized to be coincidental with immunosuppression (55). It is speculated that based on the positional role of C8G as the protein that provides a binding site for the first C9 molecule in the structural formation of the MAC pore [see (56) for an overview of MAC protein structure], the significant downregulation of C8G between T1 and T2 can likely be attributed to inhibitory CD55 or CD59 (that was not present in our proteomics analysis). Furthermore, there are additional molecules and mechanisms that may regulate MAC formation through C8G expression such as anti-apoptotic processes that promote cell survival (57). This may also explain the immunosuppressed state of post-stroke patients and infections (58), whereby the system is not able to react as efficiently to acquired pathogens such as viral pneumonia due to increased fluid phase MAC regulation (59).

### Limitations and Future Research

These results are limited to interpretation based on the sample characteristics of a cohort of mild stroke patients over a 12-month period post-stroke. During this period, it is recognized that other potential concurrent processes such as recurrent stroke, infections, venous thromboembolism could impact on the results: however, we were not able to control or adjust for such factors in the current analyses. The lack of an age matched control sample represents a challenge to external validity, and it is uncertain if these changes could also occur in people without stroke, as a process of senescence and aging or other comorbidities. Additionally, our results were not controlled for by age and baseline stroke as the analyses using the EnrichmentBrowser package did not have a streamlined function to address confounds. The profile of complement expression in this sample may also have been influenced by the myriad of post-stroke medications that currently include aspirin, warfarin, and clopidergel. Although these pharmacological agents target aspects of the coagulation system, it is possible that they also exhibit regulatory effects on the complement system (60) via the intersection between hemostasis and innate immunity (61). These confounds remain difficult to statistically control in the context of bioinformatics models, and should form part of the conceptualization in the design phase in future studies (62). While the focus of the current study was to explore evidence of change in biological pathways over the first-year post-stroke, it is recommended that future studies investigate the relationship between changes in these pathways and changes with age as well as in neurological and functional recovery profiles.

The sample preparation methods used in this study did not include fractionation or depletion of the plasma samples, leading to a low number of proteins identified relative to the blood proteome. Blood samples represent a very complex sample with high dynamic range for proteomics analysis, with a proportionally high abundance of proteins such as albumin, haptoglobin, fibrinogen, and immunoglobulin G (63). Future studies could increase the range of identified proteins by depleting abundant plasma proteins with various methods before MS analysis (64, 65); although some popular depletion columns also remove proteins identified here as differentially expressed such as those within the complement system (66). In addition to this, limitations to the discovery approach imply that there is a degree of uncertainty in the ability to identify a complete group of proteins (67, 68), especially where set and network-based bioinformatics are the intended analyses. Therefore, after pathways are identified by robust techniques such as GGEA, the integrity of these pathways can be enhanced by examining proteins that are missing in the pathway and directly related to pathway activation and function by targeted proteomics techniques such as single or mass reaction monitoring (SRM or MRM) in MS with immunoaffinity enrichment (69).

In this study, the LC-MS methodology did not detect the specific formation of C3 or C5 chains or convertase protein complexes. Therefore, the pathway representations of relative abundance for C3 and C5 may not necessarily reflect the abundance of downstream protein chains of the active products. Specifically, C3a and C5a would be interesting proteins to target as they have potent effects on inflammation physiology and would be of concern especially if shown to be elevated at 3 months post-stroke. Fluid phase identification of inactive pre-MAC complexes with protein S such as sC5b-7, 8 and 9 in addition to VTN and CLU may also provide further information on the regulatory activity of the MAC in stroke survivors. Furthermore, the longitudinal profile of complement in this study and others (25, 47) suggests that complement dysregulation begins in the first week post-stroke and continues for at least 3 months, and may set a post-stroke level of immune homeostasis. This finding needs to be further validated at other timepoints within this window, especially in relation to infection based mortality (70). To this extent, it is possible that the post-stroke immune profile is settled at 3 weeks to 1 month post-stroke when adaptive immunity is engaged in phagocytizing dead cells (44) and resolution of cerebral microedema (71).

## Conclusion

The biology of post-stroke recovery is not well-understood, with patients exhibiting varying profiles based on factors such as their age, location of lesion, and degree of stroke damage (72). This study aimed to characterize common peripheral biological systems involved in post-stroke recovery by examining the longitudinal proteomics profile of EDTA plasma in stroke survivors. Specifically, elements of the alternative pathway of complement system and MAC proteins, i.e., CFI, C6, C8A, C8G, C9 were found to be activated but also undergoing regulation at 3 months post-stroke. This increased turnover may lead to the upregulation of anaphylatoxins C3a and C5a that could explain the prolonged sterile inflammation profile of post-stroke survivors. These results suggest a biomechanism for post-stroke immunosuppression by complement system regulatory proteins. This knowledge may be useful to guide assessment in the clinical setting for post-stroke infections and immune recovery in the 3-month recovery window. For example, it may be used to inform future recommendations for in-hospital and post-stroke infections by suggesting clinical utility of complement panel blood tests. Future investigations should examine the clinical implications of this biological profile and determine the temporal trajectory of immune homeostasis post-stroke.

## Data Availability Statement

The datasets presented in this study can be found in online repositories. The names of the repository/repositories and accession number(s) can be found below: http://www.proteomexchange.org/, PXD015006.

## Ethics Statement

The studies involving human participants were reviewed and approved by Human Research Ethics Committee of Austin Health, all participating Hospitals and La Trobe University. The patients/participants provided their written informed consent to participate in this study.

## Author Contributions

VN, NR, SC, LC, and GH contributed to project conception, data analysis, and draft editing. PF and HR contributed to data analysis and project conception. TW, HM, DH, SD, and GD contributed to project conception. All authors contributed to the article and approved the submitted version.

## Conflict of Interest

The authors declare that the research was conducted in the absence of any commercial or financial relationships that could be construed as a potential conflict of interest.
