# Supplementary material for: Longitudinal Stroke Recovery Associated With Dysregulation of Complement System—A Proteomics Pathway Analysis
Source: Front Neurol. 2020 Jul 28;11:692. doi: 10.3389/fneur.2020.00692 (PMC7399641; doi:10.3389/fneur.2020.00692)
Supplement: Supplementary file 1 [file Data_Sheet_1.docx]

Supplementary Methods

**Blood collection and storage**

All samples were collected in Benton-Dickson (BD) EDTA coated 4ml vacutainers and were mixed and left to stand in ambient room temperature for 30 minutes. The tubes were then centrifuged at 1100-1300 *g* at room temperature and the resulting plasma was aliquoted into cryotubes and immediately stored in a -80^o‑^C freezer. For transport from the central study freezer to the analysis site, temperature was kept at -70^o‑^C to -80^o‑^C on dry ice before transfer into a -80^o‑^C freezer.

**Sample Processing and Trypsination**

10μL of plasma from each patient was first stabilized in a prepared tube of 100μL 7M urea and 3M thiourea pH = 8.3. Samples were incubated overnight with 1μL tris-2-(carboxyethyl) phosphine (TCEP) at 22^o^C. The following day, 4μL of 2-iodoacetamide was added to each sample and incubated for 45 minutes. An 800μL mixture of 1μg/μL trypsin and 50mM tris(hydroxymethyl)aminomethane was added to each sample and incubated overnight at 37^o^C.

200μL was removed into a new tube and dried on a SpeedVac (Thermo Scientific, MA, USA) for 30 minutes. 10μL of 10% trifluoroacetic acid (TFA) was added to each sample to acidify. Samples were cleaned using stage tip pipette preparations of 3 plugs of Empore polystyrenedivinylbenzene (SBD-XC) copolymer disks (Sigma Aldrich, MO, USA) for solid phase extraction using washes of 50μL methanol, 50μL 5% acetonitrile (ACN)/0.5% TFA and elution with 50μL 85% ACN/0.5% TFA. Dry samples were resuspended in 20μL 5% ACN/0.5% TFA.

**Chromatography and Mass Spectrometry**

1µg of plasma peptides (no fractionation or protein depletion) were reconstituted in 0.1% TFA and 2% acetonitrile (ACN) and loaded at 45 ° C onto C_18_ PepMap 300 µm ID × 2 cm trapping column (Thermo-Fisher Scientific) at 10 µl/min for 6 min, and washed for 6 minutes before switching the pre-column in line with the analytical column (BioSphere C_18_, 1.9 µm, 120 Å and 75 µm ID × 40 cm, NanoSeparation). The separation of peptides was performed at 45 °C, 250 nl/min using a non-linear ACN gradient of buffer A (0.1% formic acid, 2% ACN) and buffer B (0.1% formic acid, 80% ACN), starting at 5% buffer B to 45% over 105 minutes, then 95% B for 5 min followed by an equilibration step of 15 min (0.1% formic acid, 2% ACN). Data were collected on a Q Exactive HF (Thermo-Fisher Scientific) in Data Dependent Acquisition mode using m/z 350–1500 as MS scan range at 120 000 resolution, HCD MS/MS spectra were collected for the 10 most intense ions per MS scan at 15 000 resolution. Dynamic exclusion parameters were set as follows: exclude isotope on, duration 30 s and peptide match preferred. Other instrument parameters for the Orbitrap were: MS maximum injection time 30ms with AGC target 3 × 10^6^, collision at 28% energy for a maximum injection time of 110ms with AGT target of 1 × 10^5^.

**Protein Identification**

Identification and label-free quantitation of proteins across all samples were performed using MaxQuant version version 1.6.1.0 (Ref 1) and search against all reviewed and unreviewed human proteins in the Uniprot database (Jan 2017). Common contaminants and decoys were included automatically by Andromeda. MaxQuant settings included Carbamidomethyl C as a fixed modification, Oxidation of Methionine and Acetylation of protein N-terminus as variable modifications. Up to 2 missed cleavages were allowed, and peptides were required to be at least 7 amino acids in length. False discovery rate (FDR) cut-offs for both peptides and proteins in the database search were set to 1%. Both unique and razor peptides (peptides shared by different proteins of a group) were used for quantitation with a minimum of 2 peptides including at least 1 unique peptide required to calculate a protein quantitative value. The ‘match between runs’ setting in MaxQuant was used to transfer peptide identifications from one run to another on the basis of matching retention time and mass to charge ratio.
