# Supplementary material for: Longitudinal Stroke Recovery Associated With Dysregulation of Complement System—A Proteomics Pathway Analysis
Source: Front Neurol. 2020 Jul 28;11:692. doi: 10.3389/fneur.2020.00692 (PMC7399641; doi:10.3389/fneur.2020.00692)
Supplement: Supplementary file 2 [file Data_Sheet_2.docx]

Supplementary Figures

**Supplementary Figures**

These pathway diagrams are interpreted leading from the top to bottom. Node colours display the degree of log_2_ fold change between T1 and T2, with darker colours trending towards 0 (no change) and bright green or red trending towards up-regulation or downregulation respectively. Lines between nodes (edges) display the expected relationship between the molecules, with the edge weights displaying the strength of correlation between one molecule to the other. The open arrows represent pathways that are activating (positive correlation) and closed arrows represent pathways that are inhibiting (negative correlation). Depending on the intensity of the colours on the edges, blue represents consistency or agreement and red represents inconsistency or disagreement of the expected interaction between nodes comparing the reference GRNs to our plasma protein expression.

**Supplementary Figure 1**
GGEA construction of the Reactome complement pathway with additional information from differential expression analysis between T1 (3-7 days) and T2 (3 months)


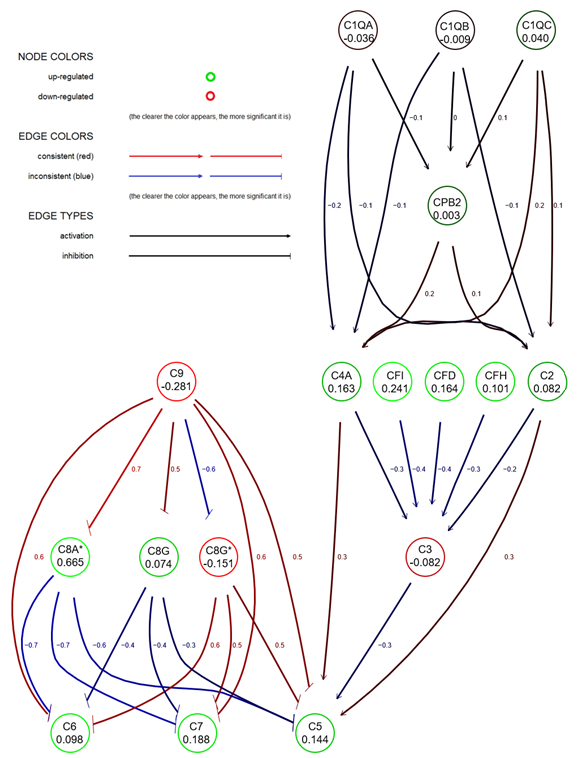


* *p* < .05 (FDR corrected)

**
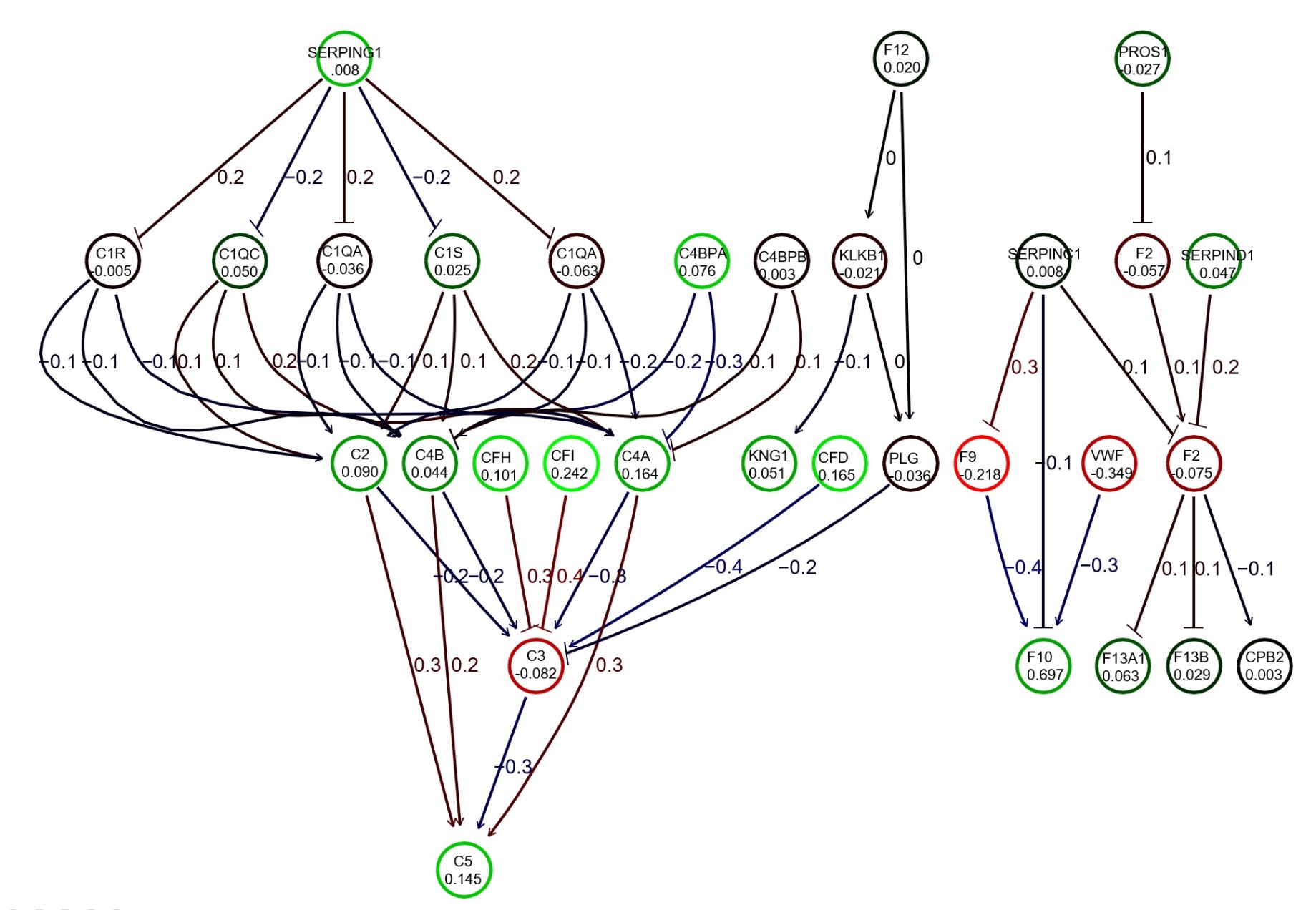
Supplementary Figure 2**
GGEA construction of the KEGG complement pathway with additional information from differential expression analysis between T1 (3-7 days) and T2 (3 months)
