# Supplementary material for: Longitudinal Stroke Recovery Associated With Dysregulation of Complement System—A Proteomics Pathway Analysis
Source: Front Neurol. 2020 Jul 28;11:692. doi: 10.3389/fneur.2020.00692 (PMC7399641; doi:10.3389/fneur.2020.00692)
Supplement: Supplementary file 5 [file Table_1.docx]

|  | Current Subset (*n* = 60) | | Full Cohort (*n* = 219) | |  |
| --- | --- | --- | --- | --- | --- |
| **Baseline** | *M^†^* | *SD* | *M* | *SD* | p |
| Age (years) | 68.00 | 14.60 | 69.85 | 13.20 | .13 |
| NIHSS | 4.70 | 4.76 | 7.99 | 6.74 | .00* |
| Heart Rate (per minute) | 74.52 | 11.06 | 76.06 | 13.83 | .37 |
| Systolic Blood Pressure (mm Hg) | 141.47 | 23.27 | 144.87 | 23.04 | .30 |
| Diastolic Blood Pressure (mm Hg) | 78.50 | 11.77 | 79.37 | 12.97 | .77 |
| **3 Month** |  |  |  |  |  |
| NIHSS | 1.18 | 2.31 | 2.54 | 4.90 | .04* |
| mRS* | 1.25 | 1.31 | 1.74 | 1.56 | .02* |
| MoCA | 25.93 | 4.57 | 24.61 | 5.92 | .13 |
| MADRS | 8.72 | 8.81 | 7.40 | 7.56 | .26 |
|  | Frequency^††^ | Percentage | Frequency | Percentage |  |
| Death | 0 | 0.00% | 21 | 9.59 | .01* |
| Lost to Follow-up | 0 | 0.00% | 8 | 3.65 | .13 |
| Withdrawal | 0 | 0.00% | 2 | 0.91 | .46 |
| Past Atrial Fibrillation | 4 | 6.7% | 50 | 22.83 | .01* |
| Hypertension | 26 | 43.3% | 124 | 56.62 | .07 |
| Lipid Disorder | 24 | 40.0% | 101 | 46.12 | .40 |
| Ischemic Heart Disease | 11 | 18.3% | 50 | 22.83 | .46 |
| Diabetes Mellitus | 9 | 15.0% | 42 | 19.18 | .46 |

**Supplementary Table 1**
Comparisons Between Current Subset for Proteomics Study (*n* = 60) and Original Full Cohort (*n* = 219)

* p < 0.05
^†^ T-tests were conducted between continuous variables.
^††^ Chi squared tests were conducted for categorical variables.
